# Innovating Quality Control and External Quality Assurance for HIV-1 Recent Infection Testing: Empowering HIV Surveillance in Lao PDR

**DOI:** 10.3390/v17071004

**Published:** 2025-07-17

**Authors:** Supaporn Suparak, Kanokwan Ngueanchanthong, Petai Unpol, Siriphailin Jomjunyoung, Wipawee Thanyacharern, Sirilada Pimpa Chisholm, Nitis Smanthong, Pojaporn Pinrod, Thitipong Yingyong, Phonepadith Xangsayarath, Sinakhone Xayadeth, Virasack Somoulay, Theerawit Tasaneeyapan, Somboon Nookhai, Archawin Rojanawiwat, Sanny Northbrook

**Affiliations:** 1 Department of Medical Sciences, Ministry of Public Health, Nonthaburi 11000, Thailand; kanokwan.n@dmsc.mail.go.th (K.N.); petai.u@dmsc.mail.go.th (P.U.); siriphailin.j@dmsc.mail.go.th (S.J.); wipawee.t@dmsc.mail.go.th (W.T.); sirilada.p@dmsc.mail.go.th (S.P.C.); s_nitis@kkumail.com (N.S.); pojaporn.p@dmsc.mail.go.th (P.P.); archawin.r@dmsc.mail.go.th (A.R.); 2 Department of Disease Control, Ministry of Public Health, Nonthaburi 11000, Thailand; ying.thiti@gmail.com; 3 National Center for Laboratory and Epidemiology (NCLE), Ministry of Health, Vientiane 01030, Laos; phonepadithxangsayarath@gmail.com (P.X.); xsinakhone@gmail.com (S.X.); virasack67@gmail.com (V.S.); 4 U.S. Centers for Disease Control and Prevention, Division of Global HIV/TB, Thailand and Laos, Nonthaburi 11000, Thailand; hqd4@cdc.gov (T.T.); hpz3@cdc.gov (S.N.); gdo0@cdc.gov (S.N.)

**Keywords:** HIV recent infection surveillance, dried tube specimens, quality control, external quality assessment

## Abstract

Quality assurance programs are critical to ensuring the consistency and reliability of point-of-care surveillance test results. In 2022, we launched Laos’ inaugural quality control (QC) and external quality assessment (EQA) program for national HIV recent infection surveillance. Our study aims to implement the first QC and EQA program for national HIV recent infection surveillance in Laos, utilizing non-infectious dried tube specimens (DTS) for quality control testing. This initiative seeks to monitor and assure the quality of HIV infection surveillance. We employed the Asante HIV-1 Rapid Test for Recent Infection (HIV-1 RTRI) point-of-care kit, using plasma specimens from the Thai Red Cross Society to create dried tube specimens (DTS). The DTS panels, including HIV-1 negative, HIV-1 recent, and HIV-1 long-term samples, met ISO 13528:2022 standards to ensure homogeneity and stability. These panels were transported from the Thai National Institute of Health (Thai NIH) to the Laos National Center for Laboratory and Epidemiology (NCLE) and subsequently shipped to 12 remote laboratories at ambient temperature. The laboratory results were electronically transmitted to Thai NIH 15 days after receiving the panel for performance analysis. The concordance results with the sample types were scored, and laboratories that achieved 100% concordance across all sample panels were considered to have satisfactorily met the established standards. Almost all laboratories demonstrated satisfactory results with 100% concordance across all sample panels during all three rounds of QC: 11 out of 12 (92%) in June, 10 out of 12 (83%) in July, and 11 out of 12 (91%) in August. The two rounds of EQA performed in June and August 2022 were satisfied by 8 out of 11 (72%) and 5 out of 10 (50%) laboratories, respectively. QC and EQA monitoring identified errors such as testing protocol mistakes and insufficient DTS panel dissolution, leading to improvements in HIV recency testing quality. Laboratories that reported errors were corrected and implemented further preventive actions. The QC and EQA program for HIV-1 RTRI identified errors in HIV recent infection testing. Implementing a specialized QC and EQA program for DTS marks a significant advancement in improving the accuracy and consistency of HIV recent infection surveillance. Continuous assessment is vital for addressing recurring issues.

## 1. Introduction

Human Immunodeficiency Virus (HIV) remains a pressing global health concern, with millions of new infections occurring annually [1]. Timely detection is imperative for effective management [2]. HIV recency testing is pivotal in distinguishing recent from long-term infections, aiding epidemiological studies and program monitoring [3]. This rising interest in integrating these assays into routine testing reflects a shifting landscape. Various methods, such as the Limiting Antigen Avidity (LAg) Assay, contribute to this testing, measuring antibody avidity to estimate time since infection [4]. The development of the LAg assay into a point-of-care test, exemplified by the Asante HIV-1 Rapid Test for Recent Infection (RTRI), highlights the critical importance of rigorous quality control in testing. Quality Control (QC) and External Quality Assessment (EQA) are indispensable for ensuring the accuracy, reliability, and standardization of HIV recent infection assays. QC focuses on internal processes, ensuring day-to-day testing procedures adhere to predefined standards, while EQA, conducted externally by independent organizations, assesses laboratory performance against established benchmarks [5]. These processes work together to maintain the quality of testing procedures across various laboratories [6].

In response to the specific needs of the Lao People’s Democratic Republic (Lao PDR), we introduced a pioneering quality control program for HIV recent infection testing utilizing dried tube specimens (DTS). This initiative not only addresses local requirements but also contributes valuable insights to the global dialogue on enhancing diagnostic precision. Dried tube specimens represent a transformative approach to sample collection, offering stability, ease of transport, and a low-cost alternative. The implementation of a tailored QC and EQA program for DTS signifies a significant advancement in the accuracy and consistency of recent infection testing.

We present a model for other countries facing similar challenges, promoting collaboration and innovation in HIV testing practices. By describing our experiences and findings, we aim to inspire progress and contribute to the ongoing efforts in QC and EQA programs for recency surveillance worldwide.

## 2. Materials and Methods

### 2.1. Source of HIV Samples and Characterization

The preparation of HIV specimens was conducted in Thailand, and the testing procedure adhered to the Thailand National Guidelines algorithm. HIV plasma samples preserved in acid citrate dextrose anticoagulant were obtained from de-identified blood units that were rejected and collected by the Thai Red Cross Society, ensuring the confidentiality of the donors. Initially, the plasma specimens underwent screening using Elecsys HIV Combi PT (Roche Diagnostics Ltd., Mannheim, Germany), followed by confirmation with Serodia HIV-1/2 MIX (Fujirebio Inc., Tokyo, Japan) and Alere HIV Combo (Alere Medical Co., Ltd., Chiba, Japan), following the manufacturers’ instructions.

To further characterize HIV specimens, they were tested using the HIV-1 RTRI, Asanté™ HIV-1 Rapid Recency^®^ Assay (Sedia Biosciences Corporation, Beaverton, OR, USA). Three samples were tested in triplicate to differentiate between negative, recent, and long-term infections, following the protocol outlined in the Asanté™ HIV-1 Rapid Recency^®^ Assay, HIV-1 RTRI package insert.

### 2.2. Ethical Considerations

Our activity was conducted in compliance with ethical standards and was approved by the Research Ethics Committee of the Department of Medical Sciences, Thailand Ministry of Public Health (Study code 12/2565). It was deemed to be a surveillance activity by the Thai Ministry of Public Health Ethics Committee and was deemed not research and was conducted under applicable federal law under CDC policy.

### 2.3. Preparation of DTS for HIV-1 RTRI

Plasma samples with HIV-1 RTRI-recent and long-term positive results underwent inactivation at 56 °C for 30 min, while HIV-1 seronegative samples remained untreated. The preparation of DTS followed the method described by Parekh et al. [5], with the addition of green food coloring at a concentration of 0.001% for straightforward visual indication. Each sample was aliquoted into individual tubes (20 µL each) and left to dry at room temperature in a biosafety cabinet overnight or until completely dried. For the project, DTS sample panels were prepared, consisting of 72 HIV-1 RTRI recent, 72 HIV-1 RTRI long-term, and 60 HIV-1 negative samples. Each DTS sample was subsequently subjected to triplicate testing for characterization, homogeneity, and stability. The DTS were reconstituted with 200 µL of PBS-0.05% Tween before HIV-1 RTRI testing.

### 2.4. Homogeneity and Stability of DTS QC and EQA for HIV-1 RTRI

To ensure the consistency of all prepared HIV DTS, a homogeneity test was conducted, involving sampling DTS HIV-negative, HIV-1 RTRI recent, and HIV-1 RTRI long-term specimens according to ISO 13528:2022 guidelines [7]. Ten randomly selected samples from each of the three categories underwent testing with HIV-1 RTRI, and the results were meticulously scrutinized for consistency.

Stability testing of the DTS was also carried out. The samples were stored at temperatures, ranging from 20 °C to 37 °C, for 6 weeks and at 2–8 °C for 6 months. Weekly testing using the HIV-1 RTRI was conducted to evaluate the samples’ stability under varying temperature conditions.

### 2.5. Packaging and Shipping of DTS for HIV-1 RTRI

*DTS Quality Control (QC) Panel*

The DTS QC panel comprised three samples per round representing HIV-negative, HIV-1 RTRI-recent infection, and HIV-1 RTRI-long-term infection. Each panel included instructions for reconstitution and testing, along with the necessary tube reconstitution buffer (PBS-0.05% Tween). The packaged DTS QC panels were shipped at ambient temperature to the Laos National Center for Laboratory and Epidemiology (NCLE). These panels were dispatched monthly from NCLE to 12 HIV viral load laboratories in Lao PDR from June to August 2022. Upon receipt, laboratories reconstituted each DTS sample by adding 200 μL of PBS-0.05% Tween. The mixture was thoroughly mixed and allowed to dissolve for 5–10 min at room temperature. Subsequently, the reconstituted DTS samples were tested using the Asanté™ HIV-1 Rapid Recency^®^ Assay.

*DTS External Quality Assessment (EQA) Panel*

Similar to the DTS QC panels, the DTS EQA panels were shipped to NCLE at room temperature along with tube reconstitution buffer (PBS-0.05% Tween) and instructions for reconstitution. The EQA panel comprised four samples per round representing HIV-negative, HIV-1 RTRI-recent infection, and HIV-1 RTRI-long-term infection statuses. These panels were dispatched to 11 HIV viral load laboratories in June (two samples of HIV-1 RTRI-long-term, one sample of HIV-1 RTRI-recent, one sample of HIV-1 negative) and in August 2022 (one sample of HIV-1 RTRI-long-term, two samples of HIV-1 RTRI-recent, one sample of HIV-1 negative).

### 2.6. Evaluating the Performance of DTS QC and EQA for HIV-1 RTRI

*Data Collection*

The QC and EQA panels were shipped to 12 and 11 HIV viral load laboratories across Lao PDR, respectively. The 12 laboratories participating in this program were selected based on their operational roles within Lao PDR’s national HIV testing and surveillance system. These laboratories routinely conduct HIV viral load testing and/or implement HIV-1 rapid tests for recent infection (RTRI) under national guidelines.

The DTS QC panel included three samples per round, while the EQA panel comprised four samples per round, following WHO recommendations for pilot studies in resource-limited settings. The panel sizes were designed to allow detection of critical errors in specimen classification (HIV-negative, recent infection, long-term infection) while remaining feasible for implementation given resource and logistical constraints. This pilot approach provided an initial assessment of laboratory performance and established a foundation for potential future program expansions.

QC results were collected during June, July, and August 2022, and EQA performance was monitored in June and August 2022. Each laboratory was required to electronically submit their testing results to the Thai National Institute of Health (Thai NIH) within 15 days of receiving the sample panels.

*Performance Criteria and Statistical Methods*

Descriptive statistics were used to summarize laboratory performance in both the QC and EQA programs. Pass rates were expressed as counts and percentages with 95% confidence intervals (CIs), calculated using the exact Clopper-Pearson method.

To assess whether there were significant differences in pass rates across rounds, Cochran’s Q test was used for QC rounds (paired binary data across more than two rounds). Fisher’s exact test was used for comparing EQA pass rates between two rounds. A *p*-value < 0.05 was considered statistically significant.

*Evaluation Protocol*

Laboratories that achieved 100% concordance were considered to have met the established standards. In cases where performance fell below 100%, a detailed investigation into the primary cause of discrepancy was initiated. The investigation process involved close monitoring, thorough analysis of testing procedures, and identification of areas for improvement. Additionally, all laboratories with discordant results received written feedback, and summary findings were discussed in post-EQA meetings to prevent recurrence.

## 3. Results

### 3.1. DTS Characterization

Plasma specimens tested with Elecsys HIV Combi PT (Roche Diagnostics Ltd., Mannheim, Germany), Serodia HIV-1/2 MIX (Fujirebio Inc., Tokyo, Japan), and Alere HIV Combo (Alere Medical Co., Ltd., Chiba, Japan) showed concordance across all tests, demonstrating that the drying process did not affect the HIV serostatus (Table 1).

### 3.2. Homogeneity and Stability of DTS for HIV-1 RTRI

The homogeneity results for 10 of each of the HIV-1 negative, HIV-1 RTRI-recent infection, and HIV-1 RTRI-long-term infection samples indicated consistency, suggesting uniformity in the prepared DTS samples (Table 2). The short-term stability study showed that DTS for HIV-1 RTRI remained stable at room temperatures ranging from 20 °C to 37 °C for 6 weeks. Additionally, stability was maintained for 6 months when stored at 2–8 °C, as depicted in Table 2.

### 3.3. QC Monitoring Performance

QC discrepancies were as follows: June 2022: LAB07 misclassified a QC-negative sample as long-term and a QC-long-term sample as negative; July 2022: LAB03 misclassified a QC-negative sample as recent, LAB10 misclassified a QC-long-term sample as recent; and August 2022: LAB02 misclassified a QC-negative sample as recent (Table 3). All 12 HIV viral load laboratories (100%) participated in QC monitoring from June to August 2022. The monthly pass rates were as follows: June 2022: 91% (11/12 laboratories; 95% Confidence Interval [CI]: 59.8–99.6%); July 2022: 83% (10/12 laboratories; 95% CI: 51.6–97.9%); and August 2022: 91% (11/12 laboratories; 95% CI: 59.8–99.6%). Cochran’s Q test revealed no statistically significant difference in pass rates across the three months (Q = 1.33, *p* = 0.513), indicating stable laboratory performance over time (Table 5).

### 3.4. EQA Performance

The participation rates for EQA testing were 100% (11/11 laboratories) in June and 91% (10/11 laboratories) in August. EQA pass rates were as follows: June 2022: 72% (8/11 laboratories; 95% CI: 39.0–94.0%); and August 2022: 50% (5/10 laboratories; 95% CI: 18.7–81.3%). Fisher’s exact test showed no statistically significant difference in pass rates between June and August (*p* = 0.361), although there was a decline in performance in August. EQA discrepancies included the following: June 2022: LAB01 and LAB10 misclassified long-term samples as recent, and LAB07 reported both a false recent result on a long-term sample and an invalid result on a recent sample; and August 2022: five laboratories (LAB05, LAB06, LAB07, LAB08, and LAB11) failed to meet performance criteria (Table 4 and Table 5).

## 4. Discussion

This study introduced Lao PDR’s first national quality control (QC) and external quality assessment (EQA) program using noninfectious dried tube specimen (DTS) panels for HIV-1 recent infection surveillance. The program aimed to strengthen laboratory capacity and improve the reliability of recent infection testing. While most laboratories demonstrated satisfactory QC performance, lower pass rates in EQA rounds highlighted areas for improvement and capacity building.

QC panels were distributed in June, July, and August 2022 to monitor testing consistency and accuracy. Performance results showed pass rates of 91% in June, 83% in July, and 91% in August. Laboratories with unsatisfactory results were advised to investigate root causes and implement corrective actions. Common errors included sample mislabeling, incorrect test procedures, data entry mistakes, and misinterpretation. For invalid results, retesting with new kits was required to identify procedural or kit-related issues.

EQA panels were distributed in June and August 2022 to assess broader laboratory performance. Despite full participation, the pass rate declined from 72% in June to 50% in August, reflecting the complexity of standardized inter-laboratory testing. Analytical and procedural discrepancies were identified, including false recent results and invalid outcomes. Laboratories with unsatisfactory results underwent structured root-cause investigations involving documentation review, site communication, corrective action guidance, and follow-up monitoring. They were required to resolve deficiencies and demonstrate corrective measures in subsequent rounds.

These findings reinforce the essential role of QC and EQA in supporting HIV recency testing and surveillance. Rapid recency testing enables near real-time monitoring of HIV transmission, particularly among high-risk populations. When integrated with epidemiological data, such testing facilitates more targeted and effective interventions. Early identification of recent HIV infections is critical for timely treatment initiation, reducing transmission, and improving health outcomes [1,2,8]. Additionally, recency assays play a pivotal role in estimating HIV incidence and understanding transmission dynamics. WHO guidelines emphasize the importance of robust quality assurance systems including QC and EQA to ensure testing accuracy, integrity, and comparability [9,10,11].

This study also demonstrates the feasibility and practicality of using DTS for HIV-1 recent infection testing. DTS offers key logistical advantages over liquid specimens, including ambient stability (up to 37 °C for 6 weeks), elimination of cold-chain requirements, simplified transport and packaging, and reduced handling risks [12,13,14,15]. These features make DTS particularly well suited for remote and resource-limited settings.

However, successful implementation requires consideration of long-term sustainability. Potential challenges include variability in DTS quality, adaptation to different laboratory infrastructures, and maintaining supply chain resilience. Furthermore, cost factors including production, training, and distribution must be addressed during program design to ensure scalability and continuity. In Lao PDR, technical assistance and training were critical for achieving accurate testing and reporting. Therefore, future adaptations of this model in other countries should integrate dedicated training, mentorship, and technical support.

Overall, this DTS-based QC and EQA program provides a viable model for strengthening HIV testing quality in low-resource settings. It offers a scalable, affordable, and practical solution for enhancing HIV surveillance and diagnostic capacity, supporting global efforts toward the UNAIDS 95-95-95 targets by 2030. The lessons from Lao PDR can inform similar initiatives in other countries seeking to improve HIV quality assurance frameworks.

## 5. Conclusions

Ensuring the accuracy of recent HIV test results is crucial for effective public health strategies. The implementation of a tailored QC and EQA program for dried tube specimens (DTS) represents a significant advancement in recent infection testing accuracy. We address this imperative by introducing a pioneering quality control program in Laos PDR for HIV recent infection testing, acknowledging the unique challenges and opportunities presented by the use of DTS, which has implications for public health in Laos and other resource-limited countries. The DTS QC and EQA sample panels were conveniently transported at room temperature and did not require regulation for shipping infectious substances, making them suitable for EQA programs in rural and remote areas. Identifying significant errors emphasizes the urgent need to enhance HIV recency testing, particularly in remote areas. Ongoing review of laboratory performance will ensure that identified problems are addressed to prevent consistent issues from recurring.

## Figures and Tables

**Table 1:** Characterization results of prepared HIV DTS samples panel.

DTS Panel	Anti-HIV Test Results			HIV-1 RTRI Results			
	Elecsys HIV Combi PT	Serodia HIV-1/2 MIX	Alere HIV Combo	Asanté™ HIV-1 Rapid Recency^®^ Assay			
	Interpretation *	Interpretation	Interpretation	C	V	LT/R	Interpretation
HIV-1 Recent (*n* = 3)	Reactive	Reactive	Reactive	+	+	−	Recent
HIV-1 Long-term (*n* = 3)	Reactive	Reactive	Reactive	+	+	+	Long-term
HIV Negative (*n* = 3)	Non-reactive	Non-reactive	Non-reactive	+	−	−	Negative

* Reactive; COI ≥ 1.0, non-reactive; COI < 1.0. C; control line, V; verification line, LT/R; long term/recent line; +, Visible color observed; −, No visible color observed.

**Table 2:** Homogeneity and stability results of HIV DTS samples panel.

Sample Types	Testing Result	Homogeneity	Stability (RT 20–37 °C)	Stability (2–8 °C for 6 Months)					
			6 Weeks	1	2	3	4	5	6
HIV-1 Negative (*n* = 10)	Negative	Consistent	Negative	Negative	Negative	Negative	Negative	Negative	Negative
HIV-1 Recent (*n* = 10)	Recent	Consistent	Recent	Recent	Recent	Recent	Recent	Recent	Recent
HV-1 Long-term (*n* = 10)	Long-term	Consistent	Long-term	Long-term	Long-term	Long-term	Long-term	Long-term	Long-term

RT: room temperature.

**Table 3:** QC monitoring from 12 participating laboratories in Lao PDR, June–August 2022.

Laboratory	June-2022/Sample Type			Jule-2022/Sample Type			August-2022/Sample Type		
	HIV-1 Negative	HIV-1 Recent	HIV-1 Long Term	HIV-1 Negative	HIV-1 Recent	HIV-1 Long Term	HIV-1 Negative	HIV-1 Recent	HIV-1 Long Term
LAB01	Negative	Recent	Long term	Negative	Recent	Long term	Negative	Recent	Long term
LAB02	Negative	Recent	Long term	Negative	Recent	Long term	Recent	Recent	Long term
LAB03	Negative	Recent	Long term	Recent	Recent	Long term	Negative	Recent	Long term
LAB04	Negative	Recent	Long term	Negative	Recent	Long term	Negative	Recent	Long term
LAB05	Negative	Recent	Long term	Negative	Recent	Long term	Negative	Recent	Long term
LAB06	Negative	Recent	Long term	Negative	Recent	Long term	Negative	Recent	Long term
LAB07	Long term	Recent	Negative	Negative	Recent	Long term	Negative	Recent	Long term
LAB08	Negative	Recent	Long term	Negative	Recent	Long term	Negative	Recent	Long term
LAB09	Negative	Recent	Long term	Negative	Recent	Long term	Negative	Recent	Long term
LAB10	Negative	Recent	Long term	Negative	Recent	Recent	Negative	Recent	Long term
LAB11	Negative	Recent	Long term	Negative	Recent	Long term	Negative	Recent	Long term
LAB12	Negative	Recent	Long term	Negative	Recent	Long term	Negative	Recent	Long term
Sample passed	11/12	12/12	12/12	11/12	12/12	11/12	11/12	12/12	12/12

**Table 4:** EQA HIV-1 RTRI performance by participating laboratories in Lao PDR, June-August 2022.

Laboratory	June 2022/Sample Type				August 2022/Sample Type			
	R651-1	R651-2	R651-3	R651-4	R652-1	R652-2	R652-3	R652-4
	HIV-1 Negative	HIV-1 Long Term	HIV-1 Long Term	HIV-1 Recent	HIV-1 Long Term	HIV-1 Recent	HIV-1 Negative	HIV-1 Recent
LAB01	Negative	Recent	Recent	Recent	Long term	Recent	Negative	Recent
LAB02	Negative	Long term	Long term	Recent	not report	not report	not report	not report
LAB03	Negative	Long term	Long term	Recent	Long term	Recent	Negative	Recent
LAB04	Negative	Long term	Long term	Recent	Long term	Recent	Negative	Recent
LAB05	Negative	Long term	Long term	Recent	Long term	Recent	Long term	Recent
LAB06	Negative	Long term	Long term	Recent	Long term	Negative	Negative	Recent
LAB07	Negative	Recent	Long term	Invalid	Long term	Recent	Long term	Negative
LAB08	Negative	Long term	Long term	Recent	Recent	Negative	Negative	Recent
LAB09	Negative	Long term	Long term	Recent	Long term	Recent	Negative	Recent
LAB10	Negative	Recent	Recent	Recent	Long term	Recent	Negative	Recent
LAB11	Negative	Long term	Long term	Recent	Negative	Long term	Long term	Recent
Sample passed	11/11	8/11	9/11	10/11	8/10	7/10	7/10	9/10

**Table 5:** Overall QC and EQA monitoring performance.

Round	Participation Rate	Pass Rate (*n*, %)	95% CI	Significant Errors (Laboratories)
**QC-June 2022**	12/12 (100%)	11/12 (91%)	59.8–99.6%	LAB07 misclassified negative and long-term samples
**QC-July 2022**	12/12 (100%)	10/12 (83%)	51.6–97.9%	LAB03 (false recent on negative sample) LAB10 (false recent on long-term sample)
**QC-August 2022**	12/12 (100%)	11/12 (91%)	59.8–99.6%	LAB02 (false recent on negative sample)
**EQA-June 2022**	11/11 (100%)	8/11 (72%)	39.0–94.0%	LAB01, LAB10, LAB07 (multiple errors, incl. invalid results)
**EQA-August 2022**	10/11 (91%)	5/10 (50%)	18.7–81.3%	LAB05, LAB06, LAB07, LAB08, LAB11 (failed performance)

Statistical tests: Cochran’s Q test for QC pass rate trends across 3 months; Fisher’s exact test for EQA pass rates between rounds.

## Data Availability

The data are unavailable due to privacy concerns.
